# Metabolite Profile and In Vitro Beneficial Effects of Black Garlic (*Allium sativum* L.) Polar Extract

**DOI:** 10.3390/nu13082771

**Published:** 2021-08-13

**Authors:** Paola Bontempo, Paola Stiuso, Stefania Lama, Assunta Napolitano, Sonia Piacente, Lucia Altucci, Anna Maria Molinari, Luigi De Masi, Daniela Rigano

**Affiliations:** 1 Department of Precision Medicine, University of Campania Luigi Vanvitelli, Via L. De Crecchio 7, 80138 Naples, Italy; paola.stiuso@unicampania.it (P.S.); stefania.lama@unicampania.it (S.L.); lucia.altucci@unicampania.it (L.A.); annamaria.molinari@unicampania.it (A.M.M.); 2 Department of Pharmacy, University of Salerno, via Giovanni Paolo II 132, 84084 Fisciano, Italy; anapoli@unisa.it (A.N.); piacente@unisa.it (S.P.); 3 National Research Council (CNR), Institute of Biosciences and BioResources (IBBR), Via Università 133, 80055 Naples, Italy; 4 Department of Pharmacy, University of Naples Federico II, Via Domenico Montesano 49, 80131 Naples, Italy

**Keywords:** black garlic, methanol extract, LC-ESI/LTQOrbitrap/MS/MS, secondary metabolites, cellular bioactivity, cancer cells

## Abstract

Over the centuries, humans have traditionally used garlic (*Allium sativum* L.) as a food ingredient (spice) and remedy for many diseases. To confirm this, many extensive studies recognized the therapeutic effects of garlic bulbs. More recently, black garlic (BG), made by heat-ageing white garlic bulbs, has increased its popularity in cuisine and traditional medicine around the world, but there is still limited information on its composition and potential beneficial effects. In this study, the metabolite profile of methanol extract of BG (BGE) was determined by high-performance liquid chromatography coupled to tandem mass spectrometry in high-resolution mode. Results allowed to establish that BGE major components were sulfur derivatives, saccharides, peptides, organic acids, a phenylpropanoid derivative, saponins, and compounds typical of glycerophospholipid metabolism. Characterization of the BGE action in cancer cells revealed that antioxidant, metabolic, and hepatoprotective effects occur upon treatment as well as induction of maturation of acute myeloid leukemia cells. These results are interesting from the impact point of view of BG consumption as a functional food for potential prevention of metabolic and tumor diseases.

## 1. Introduction

Nowadays, plants are well known to produce a species-specific array of small molecules, i.e., secondary metabolites, which have the potential to contribute to the maintenance of health status as well as to prevent, delay, and reduce a number of chronic diseases and health concerns in humans [1,2]. In vitro, in silico, and in vivo studies have revealed the potential beneficial health effects of these phytochemicals and their capacity to improve human wellness and health in terms of prevention and treatment of aging-associated diseases and metabolic disorders, affecting more than 25% of the global adult population [3,4,5]. Among the natural plant products, common garlic (*Allium sativum* L.) is used worldwide as spice, medicinal plant, and vegetable having a key role in the traditional diets with a history of thousand years. It is an important species belonging to the genus *Allium* (with about 500 species), within the monocotyledonous family Amaryllidaceae (ex Alliaceae), mostly formed by bulb or corm-forming herbaceous plants [6]. Of note, the regular consumption of common garlic was shown to reduce arterial hypertension, blood levels of cholesterol, and coronary heart disease; further evidence suggest that garlic also has antibiotic and anticancer properties [6]. The extensive health benefits as well as sensory properties of garlic are bound to the broad variety of its secondary metabolites, such as saponins, phenolics, and sulfur derivatives, which are typically alk(en)yl sulfoxides of the amino acid cysteine [6]. One of the main organosulfur compounds responsible for the typical smell of garlic is the diallyl thiosulfinate allicin, formed from the colorless and tasteless sulfoxide alliin through the action of the alliinase enzyme when garlic cloves are macerated or crushed [7]. Garlic is also rich in minerals and microelements such as magnesium, calcium, phosphorus, iodine, and iron, with traces of zinc, manganese, selenium, and some vitamins [8,9].

It was only in relatively recent times that the characteristic smell and taste of fresh garlic were eliminated through a maturation process that allows to obtain the so-called black garlic (BG), originated in South-East Asia and quickly spread to the United States of America, before arriving in Europe [8,9,10]. Although it has only been introduced in western countries in the last two decades, it is receiving attention among the natural remedies for its bioactive ingredients. BG is so called because the color of the fresh garlic bulbs after the production process, which takes place at controlled high temperature and humidity for about 1 month, changes to the typical dark brown of bread crust, coffee, and cocoa, as well as the taste to sweet and the consistency to chewy. Chemical transformations take place, such as reactions of Maillard and enzymatic browning, producing the pigments melanoidins, which confer high nutritional value by exerting radical-scavenging activity [11,12]. The molecules responsible for the flavor typical of fresh garlic (e.g., allicin) are transformed into the antioxidants S-allyl cysteine (SAC) and S-allylmercaptocysteine (SAMC) [8,9]. Due to the reduction of the allicin content, unlike the fresh product, BG does not release pungent aromas. Thus, the appreciated properties of BG are mainly due to new chemical compounds, although many of them are not yet fully known, as not enough studies have been carried out.

BG made by heat-ageing white garlic bulbs has now been introduced into the high-level cuisine and traditional medicine [10], also due to its polyphenols, sugars, and volatiles that originate after processing [13]. Different studies showed appreciated beneficial effects and indicated that the bioactive compounds of BG are useful in disease prevention and treatment [8,9]. According to the recent scientific literature, the BG extracts showed, among others, anti-inflammatory, anti-allergic, antioxidant, anti-diabetic, anti-obesity, hepatoprotective, and anti-cancer effects [14,15,16]. The outstanding medicinal effects of garlic on health have been extensively examined in animals as well as in humans [6], but they are not yet fully understood and consolidated for BG, which, in some cases, seems to show biological activities significantly higher than those of fresh garlic [17,18]. Based on these indications, there is a need for studies focused on demonstrating the nutritional role of BG in the modulation of metabolic and functional activities related to human health. To this aim, the present research focused on understanding the chemical composition of BG methanol extract (BGE) and its potential mechanisms of action in vitro. Methanol as extracting solvent was chosen for our study as it was showed to yield the highest amounts of metabolites [13]. The main goals of the work were: (i) to identify the metabolites occurring in BGE through liquid chromatography coupled to electrospray ionization tandem mass spectrometry in high-resolution mode (LC-ESI/HRMS/MS); (ii) to study BGE biological activities on cell model systems for the ability to induce differentiative, antioxidant, and hepatoprotective effects.

## 2. Materials and Methods

### 2.1. Chemicals

Roswell Park Memorial Institute (RPMI)-1640, bovine serum albumin (BSA), and 3-(4,5-dimethylthiazol-2-yl)-2,5-diphenyl tetrazolium bromide (MTT) were from Sigma-Aldrich (St. Louis, MO, USA). Phosphate-buffered saline (PBS) and trypsin-EDTA were purchased from Lonza (Milano, Italy). Fetal bovine serum (FBS) was from Gibco (Grand Island, NY, USA). All buffers and solutions were prepared with ultra-high-quality water. All reagents were of the purest commercial grade.

### 2.2. Plant Material and Preparation of the Methanol Extract of Black Garlic

Fermented and aged black garlic (*Allium sativum* L.) bulbs (Garlicorea) used in this study were supplied by a South Korean company (Menari Corporation, Ltd.). In brief, the bulbs (100 g) were crushed with a pestle in a mortar and then extracted in *n*-hexane (2 × 1 L, 8 h each) and CHCl_3_ (3 × 1 L, 8 h each). The sample was then carefully resuspended in methanol (solid:liquid = 1:3). The plant material was subjected to extraction with methanol three times for 1 h at room temperature in a shaker to obtain the methanol extract of black garlic (BGE) used in this study. The extracts were centrifuged (4000 g for 10 min), then the supernatants were collected and filtered by syringe filters (0.22 μm) prior to the LC-ESI/HRMS/MS analysis.

For the biological assays, after methanol evaporation, BGE was resuspended in dimethyl sulfoxide (DMSO) and used at the final concentrations indicated in each experiment.

### 2.3. LC-ESI/HRMS/MS Analysis

Qualitative analysis by liquid chromatography coupled to electrospray ionization tandem mass spectrometry in high-resolution mode (LC-ESI/HRMS/MS) was performed using a LC-ESI/HRMS system consisting of an Accela HPLC system, equipped with a quaternary Accela 600 pump and an Accela autosampler, coupled to a LTQ Orbitrap XL mass spectrometer (ThermoScientific, San Jose, CA, USA), operating in negative ionization mode. The Orbitrap mass analyzer was calibrated according to the manufacturer’s directions by using a mixture of caffeine, methionine-arginine-phenylalanine-alanine-acetate (MRFA), and Ultramark 1621 in a solution of acetonitrile, methanol, and acetic acid. Data were collected and analyzed by using the software provided by the manufacturer. The separation was carried out by using a Kinetex EVO 5.0 µm RP C18 column (Phenomenex, 150 × 2.1 mm), at a flow rate of 0.2 mL/min and a mobile phase consisting of a combination of A (0.1% formic acid in water, *v*/*v*) and B (0.1% formic acid in acetonitrile, *v*/*v*). A linear gradient from 5 to 13% B in 8 min, from 13 to 38% B in 12 min, from 38 to 46% B in 8 min, held to 46% B for 10 min, from 46 to 53% B in 7 min, held to 53% B for 15 min, was used. The autosampler was set to inject 10 µL of extract (0.5 mg/mL).

In the negative ion mode, the following experimental conditions for the ESI source were adopted: sheath gas at 15 (arbitrary units), auxiliary gas at 5 (arbitrary units), source voltage at 3.5 kV, capillary temperature at 280 °C, capillary voltage at −48 V, and tube lens at −176.47 V.

The mass range was from 100 to 1400 *m/z* with a resolution of 30,000. In order to obtain structural information, data-dependent experiments were performed by acquiring tandem mass spectra of the first and the second most intense ions from the HRMS scan event. A normalized collision energy at 30%, a minimum signal threshold at 250, and an isolation width at 2.0 were used.

### 2.4. Cell Lines and Culture Conditions

Human hepatocellular carcinoma cell line HepG2 (ATCC HB-8065) from American Type Culture Collection (ATCC, Manassas, VA, USA) was cultured in RPMI-1640 medium with 10% heat-inactivated FBS, 100 U/mL penicillin, 100 μg/mL streptomycin, and 1% L-glutamine. The cell cultures were incubated at 37 °C in a 5% CO_2_ and 95% air humidified atmosphere. Then, cells were treated using the different amounts of BGE, as reported for each experiment performed in duplicate.

Human histiocytic lymphoma cell line U937 (ATCC CRL-1593.2) was obtained from ATCC and human acute promyelocytic leukemia cell line NB4 was provided by Michel Lanotte (INSERM U-496, Centre G. Hayem Hôpital Saint-Louis, Paris, France). Cells were grown at 37 °C in 5% CO_2_ atmosphere in RPMI-1640 medium (Gibco, NY, USA), supplemented with 10% heat-inactivated fetal bovine serum (FBS), 1% L-glutamine, 1% ampicillin or streptomycin, and 0.1% gentamicin. Cells were plated at 1000 cells/mL density and then treated using the different amounts of BGE, as reported for each experiment performed in triplicate.

### 2.5. Cellular Bioactivity

#### 2.5.1. Cell Viability Assay

HepG2 and U937 cells were plated in 96-well plates at the seeding density of 5 × 10^3^ cells/well in RPMI complete medium. After 4 h incubation at 37 °C, the cells were treated for 24 h with different concentrations of black garlic extract (BGE, 0–2.50 mg/mL). Then, cell viability was evaluated by adding 3-(4,5-dimethylthiazol-2-yl)-2,5-diphenyltetrazolium bromide (MTT) solution in PBS to a final concentration of 5 mg/mL, as previously described [19,20]. The culture medium was removed after 4 h of incubation at 37 °C, and the purple formazan crystals were solubilized by mixing for 5 min with 150 μL dimethyl sulfoxide (DMSO). Then, measurements of absorbance at 570 nm by Bio-Rad 550 microplate reader (Bio-Rad Laboratories, Milan, Italy) estimated cell viability inhibition, expressed as a percentage of the control. Each experiment was carried out in triplicate.

#### 2.5.2. Cellular Oxidative Stress: Nitrite Levels in the Culture Medium

Nitric oxide (NO) is rapidly converted into the stable end products nitrite and nitrate. Briefly, nitrite was measured by the Griess reaction using 10 μL of medium mixed with an equal volume of Griess reagent (0.5% sulfanilamide, 2.5% H_3_PO_4_, and 0.05% naphthyl ethylene diamine in H_2_O) and then incubated for 10 min at room temperature. Measurements of absorbance at 550 nm were compared with a standard curve of sodium nitrite [21].

#### 2.5.3. Cellular Oxidative Stress: Lipid Peroxidation in Cytosol

The cellular lipid peroxidation was evaluated determining the toxic reactive aldehydes by thiobarbituric acid reactive substances (TBARS) assay. In brief, the homogenates of U937 and HepG2 cells treated for 24 h without and with 1.25 and 2.50 mg/mL BGE, respectively, were incubated at 95 °C for 45 min with 0.5 mL of 20% acetic acid solution (pH 3.5) and 0.5 mL of 0.78% aqueous solution of thiobarbituric acid, as previously described [20,21]. After cooling and centrifugation at 4000 g for 5 min, TBARS were quantified in the supernatant fractions by absorbance at 532 nm. Each experiment was performed in duplicate.

#### 2.5.4. Metabolic Parameters

HepG2 cells were grown in high glucose (30 mM, HG) concentration medium for 72 h and total cholesterol (CHL), triglycerides (TRG), and glucose were determined using standard chemical methods of clinical laboratory.

#### 2.5.5. Oil Red O Staining

Oil Red O (ORO) staining of neutral lipids and lipid droplets was carried out as previously described [19]. Briefly, the cells, fixed by 10% formalin solution and incubated with 60% isopropanol, were stained for 10 min with ORO solution. Then, after washing the cell cultures, the dye was extracted by isopropanol and the absorbance was measured at 520 nm. Results were expressed as A_520_/cells number.

### 2.6. Microscopic Analysis

After treatment with BGE at 2.5 and 5 mg/mL, human leukemia cell lines U937 and NB4 were grown for 48 h on multi-well plates. Cell growth in three independent experiments, each performed in duplicate, was analyzed by bright-field light microscope observation. Images were captured with DMIRB inverted microscope (Leica) using N-Plan 10× objective (Leica) and a DFC 450C camera (Leica), and then analyzed with Application Suite software (Leica).

### 2.7. Differentiation Assay

Analysis of granulocytic and monocytic differentiation was conducted as previously reported [22,23,24]. In brief, U937 cells treated with and without 2.5 mg/mL of BGE were resuspended in 10 µL phycoerythrin-conjugated CD11c (CD11c-PE) (Pharmingen, San Diego, CA, USA) or 10 µL fluorescein isothiocyanate-conjugated CD14 (CD14-FITC) (Pharmingen). Controls without BGE treatment were with 10 µL PE- or FITC-conjugated mouse IgG1 (Pharmingen). After incubation at 4 °C for 30 min in the dark, samples were washed in PBS, and resuspended in 500 µL of PBS containing 2 µL of propidium iodide (PI) (Sigma-Aldrich). Analysis of samples was performed by FACS Calibur flow cytometer with Cell Quest technology (Becton Dickinson, San Diego, CA, USA). PI-positive cells were not considered in the analysis. Each data point was the average of individual experiments performed in duplicate, and standard deviation was computed.

### 2.8. Western Blot Analysis

Cell lysis of U937 was carried out as previously reported [23]. After centrifugation of cell lysate at 13,000 *g* for 30 min, protein concentration was determined and protein extract aliquots of 40 µg were electrophoresed on 12% polyacrylamide gel and blotted as previously described [23,25]. Western blotting was performed by probing with antibodies against caspase 3 (1:500; #9662; Cell Signaling Technology, Danvers, MA, USA), caspase 8 (1:500; #9746; Cell Signaling Technology), caspase 9 (1:500; #25758; Abcam, Cambridge, UK), and cleaved poly(ADP-ribose) polymerase (PARP) (1:500; #AF-600-NA; R&D Systems, Minneapolis, MN, USA). Monoclonal antibodies against glyceraldehyde-3-phosphate dehydrogenase (GAPDH) (1:500; #sc32233; Santa Cruz Biotechnology, Dallas, TX, USA) and extracellular signal-regulated kinases 1/2 (ERKs 1/2) (1:500; #sc94; Santa Cruz Biotechnology) were used for equal sample loading to normalize the target protein expression. As a positive control, the polyphenol and anthocyanin-rich extract (PAE) from *S. tuberosum* was used, as reported by De Masi et al. 2020 [23] (data not shown).

## 3. Results and Discussion

### 3.1. Characterization of Black Garlic Methanol Extract

**Figure 1:**
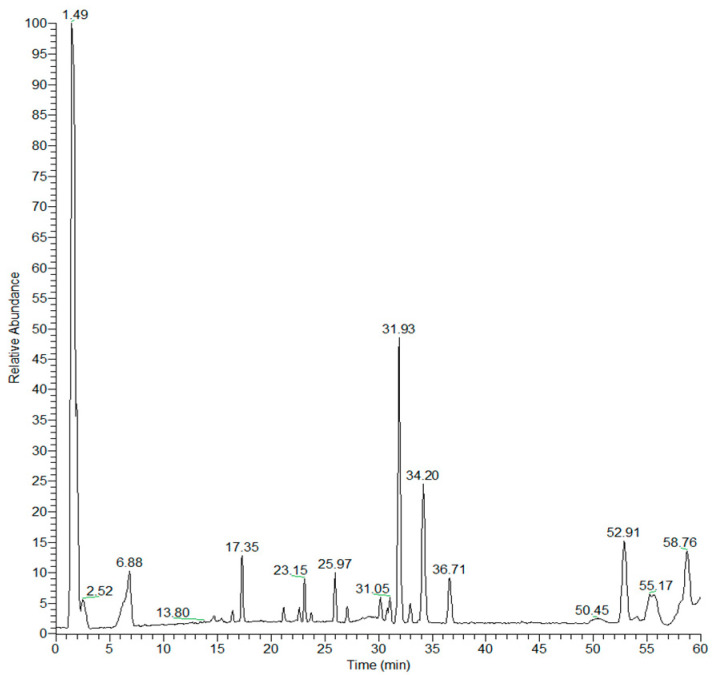
LC-ESI/HRMS chromatogram (base peak intensity, negative ion mode) of black garlic methanol extract (BGE).

In order to obtain the metabolite profile of black garlic methanol extract (BGE), an analytical approach based on high-performance liquid chromatography coupled to multiple-stage linear ion-trap and orbitrap high-resolution mass spectrometry using negative electrospray ionization mode (LC-ESI/LTQOrbitrap/MS/MS) was carried out. The analysis of LC-ESI/MS/MS spectra and the comparison with literature data allowed to assign the molecular structure of most [M-H]^−^ ions (Table 1). The LC-ESI/HRMS profile showed chromatographic peaks characterized by pseudomolecular [M-H]^−^ ions with molecular formulae suggesting the occurrence in black garlic of organosulfur compounds (**1–2**, **5**) saccharides (**3–4, 6**), peptides (**7**, **10–11**), organic acids (**8**–**9**), a phenylpropanoid derivative (**12**), saponins (**13**, **15**–**17**), and lipids (**14**, **18**–**38**) (Figure 1; Table 1).

According to literature reports and mass spectrometric data, compounds **1**, **2**, and **5** could be tentatively identified as the organosulfur compounds, characteristic of *Allium* species, S-allyl-cysteine (**1**), alliin (**2**), and allicin (**5**), belonging to the families of L-cysteine, sulfoxides, and thiosulfinates derivatives, respectively (Table 1) [13,26]. 

Compounds **3–4, 6** were identified as sugar derivatives according to their molecular formula, fragmentation pattern, and literature data. In particular, compounds **3** and **6** have been already reported in black garlic, the former as disaccharide molecule and the latter as hexose-phosphate characterized by product ions at *m/z* 79 and 97, originated by neutral loss of the hexose unit as whole or dehydrated form, and by product ions at *m/z* 199 and 169, formed by inner breakdown of the hexose moiety (Table 1) [26]. Compound **4** displayed in LC-ESI/HRMS spectrum a [M-H]^−^ pseudomolecular ion at *m/z* 323.0984, along with the [(M-H) + HCOOH]^−^ formic acid adduct ion, accounting for the molecular formula C_12_H_20_O_10_. The fragmentation pattern suggested the structure of **4** as bis-D-fructose 2′,1-2,1′-dianhydride, a disaccharide derivative already found in black garlic (Table 1) [26].

Compounds **7**, **10**, and **11** exhibited a nitrogenous molecular formula and a fragmentation behavior ascribable to peptide derivatives. In particular, according to its mass spectrometric behavior and literature data, compound **10** could be assigned as the dipeptide γ-glutamylphenylalanine, already described in black garlic (Table 1) [26]. Interestingly, compound **11** differed from **10** being 162 Da higher, as confirmed by the product ion [(M-H)-162]^−^ at *m/z* 293 occurring in its LC-ESI/MS/MS spectrum. Moreover, as for compound **10**, compound **11** yielded product ions at *m/z* 164 and 128, corresponding to the phenylalanine anion and to the dehydrated form of the glutamic acid anion, respectively, allowing to assume that **11** could be a γ-glutamylphenylalanine-*O*-hexose ester, never described before. The fragmentation pattern of **11** was also characterized by the presence of the product ion at *m/z* 290, originated by a neutral loss of 162 Da, suggesting that the hexose unit was likely esterified at the α-carboxyl group of the glutamic acid residue (Table 1). This observation guided the identification of compound **7** as L-pyroglutamic acid-*O*-hexose ester, according to its molecular formula and to the fragmentation pattern mainly characterized by the product ion at *m/z* 128, corresponding, as mentioned above, to the dehydrated form of the glutamic acid anion, i.e., the pyroglutamic form. The fragmentation pattern of **7** was further characterized by product ions at *m/z* 200 and 170, originated by inner fragmentation of the sugar unit (Table 1) [27]. Both compound **7** and **11** have been here described for the first time in black garlic.

Compound **12** showed a molecular formula and fragmentation pattern that allowed to suppose a phenylpropanoid diglycoside nature (Table 1). LC-ESI/MS/MS spectrum showed a main product ion at *m/z* 307 originated by neutral loss of 210 Da, that, according to the literature data, could be assumed as sinapyl alcohol, a phenolic compound already found in garlic [28]. Thereby, compound **12** could be tentatively supposed to be a syringin derivative, also described in *A. macrostemon* and *A. victorialis*, and precisely, a rhamnoside derivative, as supported by the presence of the product ion at *m/z* 163, likely corresponding to the dehydrated rhamnose anion (Table 1) [29].

According to literature data attesting the occurrence of saponins in *Allium* spp., compounds **15** and **16**, exhibiting consecutive neutral losses of hexose units from the [M-H]^−^ pseudomolecular ion in their LC-ESI/MS/MS spectra, could be referred as spirostanol saponins with an aglycone structure more likely corresponding to gitogenin or β-chlorogenin, already described in *A. sativum* (Table 1) [30,31,32,33]. Analogously, on the basis of its molecular formula, retention time, and fragmentation behavior, compound **17** could be tentatively assigned as tigogenin, a spirostanol aglycone already reported in *A. sativum* [30]. Finally, compound **13** could be tentatively assigned as porrigenin B 3-*O*-pentasaccharide, a spirostanone saponin found in *A. ampeloprasum*, but never reported so far in *A. sativum* [34].

The C_12_H_18_O_7_S molecular formula of compound **18** pointed to identify it as a sulfated compound that, in agreement with the fragmentation pattern and literature data, could be defined as hydroxyjasmonic acid sulfate, a stress indicator metabolite characterized by a LC-ESI/MS/MS spectrum showing a main [(M-H)-80]^−^ product ion at *m/z* 225, due to neutral loss of a SO_3_ molecule. Minor product ions at *m/z* 261 and 97, the first formed by neutral loss of a carbon dioxide molecule and indicating a carboxylic acid moiety in the parent ion, and the second corresponding to the sulfuric acid anion, were also detectable (Table 1) [35,36].

The molecular formula of compound **14** pointed to suggest it as a C_18_ trihydroxylated fatty acid, as supported by the tandem mass spectrum characterized by diagnostic product ions and neutral losses. These were generated by molecular rearrangements involving the head and the end of the acyl chain, respectively, so promptly suggesting the position on the fatty acyl chain of both hydroxyl groups and double bonds [37]. So, the observation of the product ion at *m/z* 171, corresponding to the shortened acyl chain having the carboxyl group as COO^−^ and an aldehyde end-group originated from the rearrangement of one hydroxyl function and subsequent cleavage of the C9-C10 bond, allowed to locate the OH group at C-9 (Table 1). Analogously, the product ions at *m/z* 229 and 199, originated by neutral loss of 100 and 130 Da via the same CHOH→CHO rearrangement of the hydroxyl function involving the end of the acyl chain to form a hexanal and a hydroxylated heptanal, respectively, allowed to locate the hydroxyl groups at C-12 and C-13 (Table 1). Consequently, in agreement with the assigned molecular formula, a double bond between C10-C11 carbons has to be assigned (Table 1). To our knowledge, this is the first report of oxylipin (**14)** in black garlic.

Finally, according to literature data describing the occurrence of polar lipids in garlic, metabolites belonging to glycolipid (DGMG) and lyso-phospholipid (l-PE, l-PC, and l-PI) classes could be identified (Table 1) [13,38]. On the basis of the presence in LC-ESI/MS/MS spectra of typical product ions involving the characteristic head-group of lipid molecule, the identification of 5 l-PE, 3 DGMG, 5 l-PI, and 7 l-PC species could be obtained (Table 1). More specifically, product ions at *m/z* 214 and 196 for l-PEs, at *m/z* 397 for DGMG, at *m/z* 241 for l-PI, and the [M-15]^−^ product ion formed by neutral loss of a methyl radical from the choline moiety of l-PC species (neutral loss of 60 Da from the [(M-H)+HCOOH]^−^ formic acid adduct ion) could be considered particularly significant (Table 1). The nature of fatty acid esterified on glycerol moiety of each lipid could be inferred by the check of the [RCOO]^−^ product anion in the tandem mass spectrum. The *sn*-1/*sn*-2 position of the acyl chain on the glycerol backbone, i.e., the regiospecificity, could not be determined, except in the case of couples of lyso-phospholipids composed of palmitic acid (C16:0) (**25** and **30**, **28** and **31**, **36** and **38**), for which the occurrence of both regioisomers could be asserted (Table 1).

### 3.2. Effects of Black Garlic Methanol Extract on Cancer Cell Viability and Oxidative Stress

**Figure 2:**
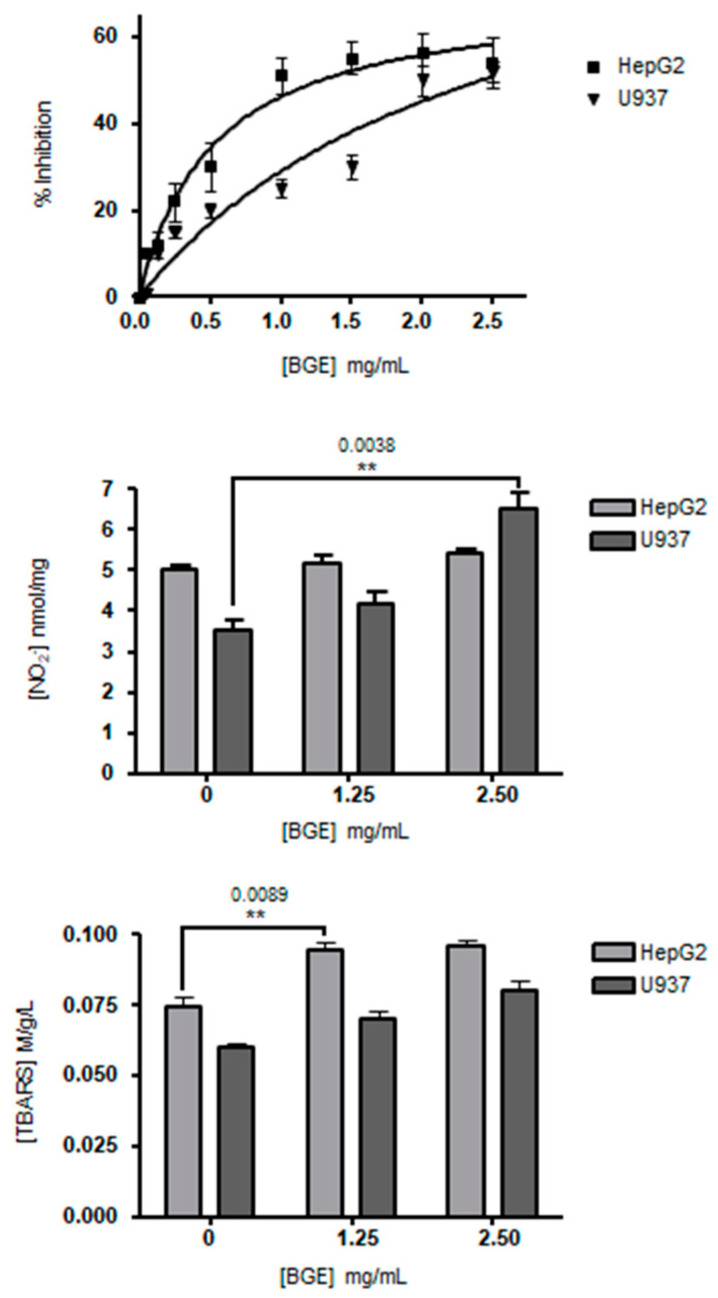
Effects of black garlic extract on cell viability and oxidative stress. In the upper panel, the inhibitory effects of different concentrations of BGE (0–2.5 mg/mL) on HepG2 and U937 cells were evaluated by MTT assay and expressed as percentage of inhibition compared to control (HepG2 and U937 cells without BGE treatment). After 24 h of BGE treatment, the IC_50_ were 0.8 mg/mL and 2 mg/mL on HepG2 and U937, respectively. In the central panel, the effect of BGE on free nitric oxide (NO) level evaluated in the intercellular medium of BGE-treated HepG2 and U937 (*p* = 0.0038) cells was reported. In the bottom panel, lipid peroxidation in cytosol was evaluated by TBARS assay in BGE-treated HepG2 (*p* = 0.0089) and U937 cells. Data were mean ± SD (*n* = 3).

The effects of black garlic methanol extract (BGE) on cell viability and oxidative stress were evaluated in vitro using human hepatocellular carcinoma (HepG2) and human leukemic (U937) cell lines. Both HepG2 and U937 cells were initially exposed to increasing BGE doses up to 2.5 mg/mL, then cell viability was assessed after 24 h by MTT assay. BGE induced dose-dependent cell viability inhibition with IC_50_ = 0.8 mg/mL and 2.0 mg/mL on HepG2 and U937 cells, respectively (Figure 2, upper panel).

The effects on cellular oxidative stress upon BGE treatments (1.25 and 2.50 mg/mL) were determined by evaluating free nitric oxide (NO) concentration as nitrites in cell culture medium and lipid peroxidation as TBARS in cytosol of both HepG2 and U937 cells (Figure 2, central and bottom panels). After 24 h of 2.50 mg/mL BGE treatment, U937 cells showed a significant increase (*p* = 0.0038) of free NO release of about 49% compared to the untreated U937, while there was no effect on HepG2 cells compared to the untreated HepG2 (Figure 2, central panel). In rats, BGE also demonstrated vasodilating effect in tail artery segments by NO induction and increased the NO release in aorta segments [39]. NO generated by the NO synthase (NOS) enzymes plays the role of intercellular messenger that regulates different key functions, such as gene expression, differentiation, cell cycle, and apoptosis [40,41,42]. The inhibition effect of BGE on U937 cells could be due to its ability of activating the production of NO by NOS enzymes and, indirectly, of scavenging superoxide anion radicals (O_2_^−^) that induce the transformation of NO into peroxynitrite (ONOO^−^), which consequently cannot play the function as a second messenger.

On the other side, the lipid peroxidation evaluated by TBARS assay in cytosol resulted in a significant increase (*p* = 0.0089) in the BGE-treated (1.25 and 2.50 mg/mL) HepG2 cells of about 25% compared to untreated cells, and to a lesser extent in U937 cells (Figure 2, bottom panel). In support of these results, previous studies demonstrated that the products of cellular lipid peroxidation act as intracellular signals able to inhibit cell proliferation and to induce differentiation [43]. Up to now, white garlic extracts have been amply demonstrated to induce ROS-dependent cytotoxicity on cancer cell lines, while only a limited number of studies showed that BGE can cause ROS-dependent cell death in cell lines [9,44]. The cytotoxic activity of saponins was shown in different cancer cell lines, such as Hep-G2, HT1080 (fibrosarcoma), HeLa (cervical cancer), HL-60 (promyelocytic leukemia), and MDA-MB-453 (breast cancer) [45]. Our data demonstrated that the BGE treatment of HepG2 cells induced antiproliferative effect and a concomitant increase of lipid peroxidation. During the fatty acid β-oxidation, the physiological collateral productions of superoxide anion radicals (O_2_^−^) and hydrogen peroxide (H_2_O_2_) trigger the self-propagating chain reactions of non-enzymatic lipid peroxidation in cells. In rat liver, Ha et al. (2015) [46] reported that the supplementation with BGE of a high fat diet led to increased expression of carnitine palmitoyltransferase-1 (CPT-1), causing TG break down to supply free fatty acid as an energy source. We think that the antiproliferative effect of BGE is most likely due to its pro-oxidant actions related to lipid peroxidation induced by an increase of fatty acid β-oxidation.

### 3.3. Black Garlic Methanol Extract Reduces Glucose, Triglycerides and Cholesterol Accumulation in Liver Cells

Hyperglycemia is known to exert injuries in liver, i.e., dysregulation of glucose, lipid, and triglyceride metabolism [47,48]. Previous studies reported that the in vivo administration of black garlic in rats led to decreased liver weights by reducing fat accumulation and decreased TBARS levels in liver, heart, and plasma [49]. HepG2 cells are an in vitro model system suitable to determine the effects of black garlic on lipid metabolism in human hepatocytes. To assess the functional role of BGE, we have grown HepG2 cells in high glucose (30 mM, HG) concentration medium for 72 h, in vitro miming the hyperglycemic effect on lipid metabolism [21,47]. Interestingly, 1.0 mg/mL BGE was significantly active in reducing glucose, cholesterol (CHO) and triglyceride (TG) cell uptake of 2.4, 3.8 and 4.9 folds, respectively, in HG-HepG2 cells compared to untreated cells (Table 2). Moreover, BGE reduced lipid peroxidation of about 1.5-fold compared to untreated HG-HepG2 cells, as evaluated by TBARS assay (Table 2).

**Figure 3:**
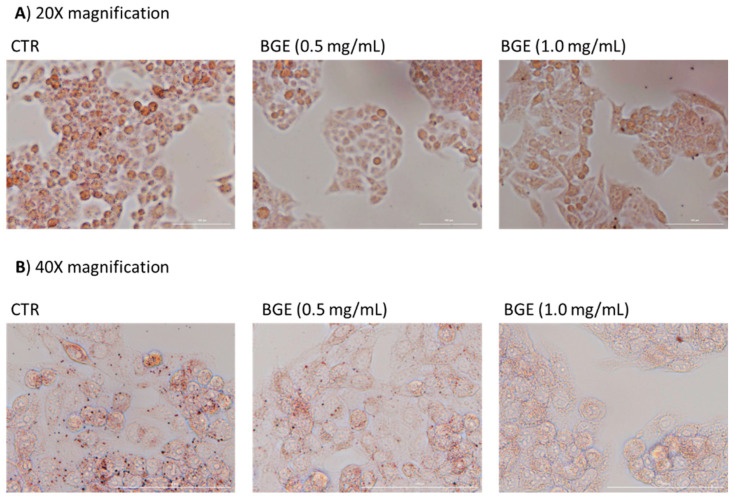
BGE reduces neutral lipid accumulation in HepG2 cells exposed to high glucose. (**A**) ORO staining microscopy at 20X magnification revealed a decrease of lipid droplets in cytoplasm of BGE-treated HG-HepG2 cells compared to untreated control cells (CTR); (**B**) lipid droplets decrease in cytoplasm of BGE-treated HG-HepG2 cells was more clearly revealed by ORO staining microscopy at 40X magnification.

Liver steatosis is the visible accumulation of lipid droplets in hepatocytes. The hypolipidemic effect of BGE was further demonstrated by neutral lipid accumulation. The HG-HepG2 cells were treated for 72 h with 0.5 and 1.0 mg/mL BGE. Oil Red O (ORO) staining method was used for the detection of intracellular lipids. ORO staining microscopy revealed a decrease of lipid droplets accumulation in cytoplasm of BGE-treated HG-HepG2 cells compared to untreated cells (Figure 3).

To quantitatively assess lipid accumulation in HG-HepG2 cells, we performed ORO colorimetric assay [21]. ORO assays on BGE-treated HG-HepG2 cells evidenced about 45% and 80% reduction of the lipid droplets at 0.5 and 1.0 mg/mL, respectively, compared to untreated cells (*p* < 0.001). Malignant cells express high levels of glucose transporters in connection with an enhanced rate of glycolysis [50]. Some studies have reported beneficial effects of BG preventing some of the cardiometabolic alterations associated with metabolic syndrome, both in humans and in experimental animals [8,9]. In our study, BGE has been shown to contain the steroidal sapogenin Tigogenin. It has been extensively reported that steroidal sapogenins from various plants have anti-obesity effects [51]. Saponins consist of an amphiphilic structure with a hydrophobic steroid or a triterpene nucleus that could form complexes with cholesterol in the lipid bilayer membrane. The formation of the saponin/cholesterol complex may be the cause of the decrease in glucose transport. Therefore, the length of treatment with BGE of HG-HepG2 cells (72 h) can decrease the absorption of glucose and consequently cause a reduction in the accumulation of neutral lipids.

Non-alcoholic fatty liver disease (NAFLD) is a chronic damage due to a dysregulated movement of lipids from adipose tissue, muscle, and gut to liver [21]. The clinical importance of NAFLD is associated with the shift towards non-alcoholic steatohepatitis (NASH), which is in turn able to evolve in liver cirrhosis and cancer, through a series of steps related to oxidative stress and excessive lipid accumulation in hepatocytes [21]. Although there is no treatment for NAFLD and NASH, natural remedies such as black garlic, able to reduce oxidative stress and lower lipid accumulation, could help to control these disorders.

### 3.4. Black Garlic Mehanol Extract Induces Differentiation in Hematological Cancer Cell Lines

Previous research showed that the aqueous extract of BG (10, 50, and 100 mg/mL) can induce antiproliferative effect and apoptosis in the human gastric cancer cells SGC-7901 [44]. Dose-dependent apoptosis was detected in the treated cells by using concentrations 10- to 20-fold higher than those used in our study. The same authors further demonstrated the anticancer ability in a tumor-bearing mouse model. Likewise, the ethanol extract of BG (20, 50, and 100 mg/mL) inhibited growth of HT29 colon cancer cells through apoptosis and cell cycle arrest [52].

**Figure 4:**
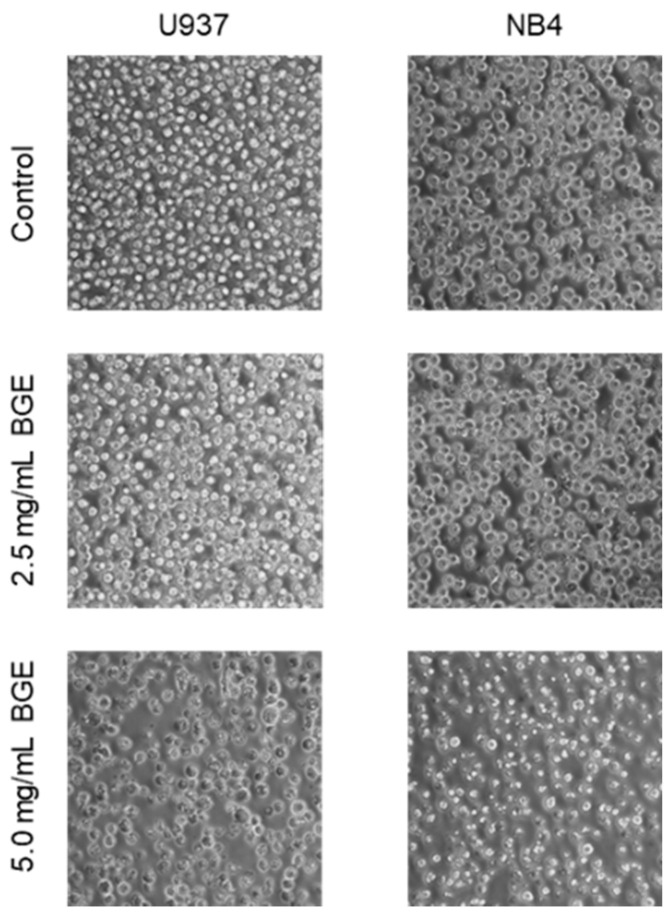
Black garlic methanol extract (BGE) induces proliferative block in hematological cancer cells. Images are representative of microscopic analysis of the indicated cell lines (U937 and NB4) after 48 h of treatment with 2.5 and 5.0 mg/mL BGE.

Then, we characterized the biological effects of the methanol extract of BG by evaluating its potential anticancer activity in myeloid cell lines. In our study, the treatment of U937 and NB4 cells with 2.5 and 5.0 mg/mL BGE for 48 h induced cell proliferation inhibition, and we also found differences in antiproliferative efficacy of BGE between the two cell lines (Figure 4). Our results showed a dose-dependent antiproliferative effect after treatment for 48 h with 2.5 and 5.0 mg/mL BGE concentrations, as clearly evidenced by microscopic analysis (Figure 4).

In our conditions, the cell line U937 was more responsive than NB4, so we selected it for further investigations on BGE effects. A greater effect was observed after BGE treatment at the concentration of 5.0 mg/mL after 48 h, as shown by the extremely reduced number of cells. Consequently, to allow for the appropriate experimental observations, the subsequent assays were performed at 2.5 mg/mL BGE for 48 h on U937 cells.

**Figure 5:**
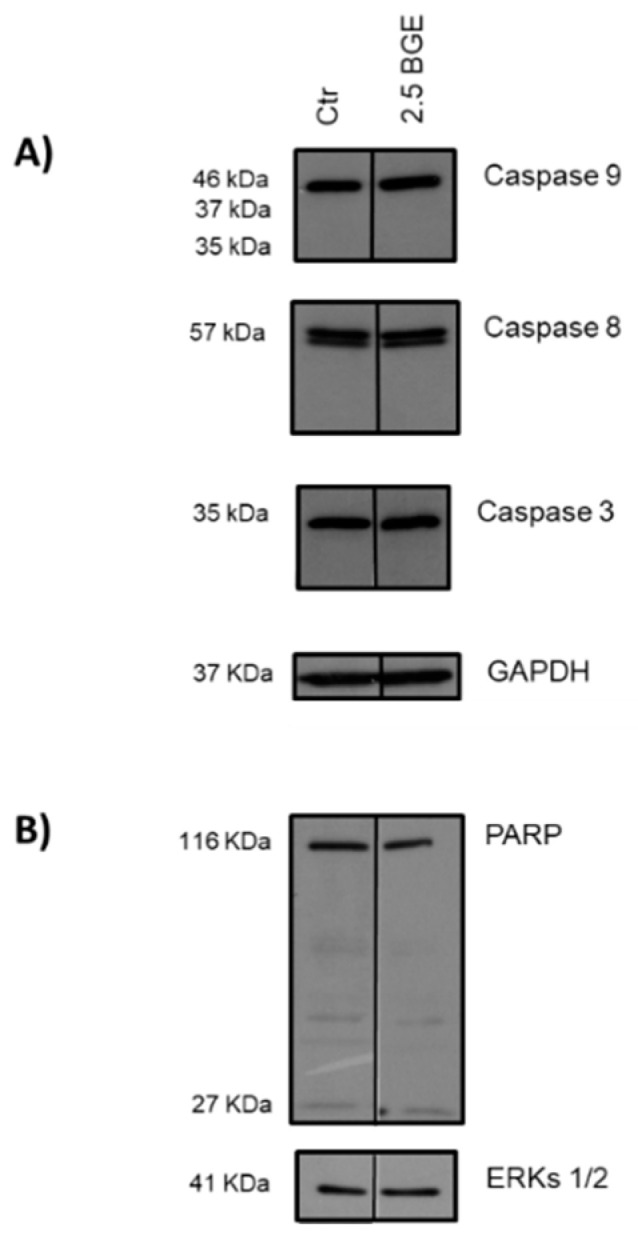
Black garlic methanol extract (BGE) did not restore the apoptotic program in U937 cells, as compared to untreated cells (Ctr). (**A**) Western blot of the indicated proteins after 2.5 mg/mL BGE treatment for 24 h on U937 cells. Glyceraldehyde 3-phosphate dehydrogenase (GAPDH) as loading control. (**B**) Western blot of the indicated protein after 2.5 mg/mL BGE treatment for 24 h on U937 cells. Extracellular signal-regulated protein kinases 1 and 2 (ERKs 1/2) as loading control.

In the same model used in our study, U937 cells, Park et al. (2014) [53] showed that the hexane extract of BG (2.5, 5, 7.5, and 10 µg/mL) inhibited cell growth. This effect was mediated, in a concentration- and time-dependent manner, by signaling cascades of both death receptor-mediated extrinsic and mitochondria-dependent intrinsic pathways of apoptosis (caspase-8, caspase-9, and caspase-3), and by cleavage of poly (ADP-ribose) polymerase (PARP), a substrate of caspase-3 considered as an apoptosis biomarker. These experiments were performed using concentrations of the hexane extract of BG with 500- to 1000-fold lower doses than those used in our study. Then, we intended to verify the presence of apoptotic effects of BGE at 2.5 mg/mL on U937 cells. The results showed that BGE does not seem to activate the caspase-dependent apoptosis pathway at 24 h post-treatment, as evidenced by the absence of cleavage of caspase-9, -8, -3, and PARP (Figure 5).

All together, our results and the literature data reported above [44,52,53] evidenced differences from 10- to 1000-fold in the biologically active doses, also depending on how the BGE is prepared (aqueous, ethanol, methanol, and hexane extract). This fact would explain why restoration of the apoptotic way was not detected with our methanol-based extract. In turn, this aspect is likely due to the different combinations of the bioactive secondary metabolites present in each extract by which the chemical composition is determined. Moreover, properties could vary from different garlic cultivars due to agronomic, genetic, and environmental factors. More recently, to confirm our results, Toledano Medina et al. (2019) [54] showed BGE to induce a decrease in leukemia cells growth, but BGE was unable to induce, consistently with the concentrations used our study, internucleosomal DNA fragmentation, a marker of the genomic integrity associated with the activation of the apoptotic program.

**Figure 6:**
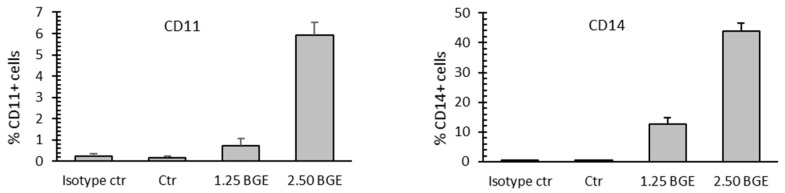
Black garlic methanol extract (BGE) induced hematological cancer cell differentiation. FACS analysis of CD11c (left) and CD14 (right) expression in U937 cells upon treatment with 1.25 and 2.50 mg/mL BGE for 24 h, as compared to untreated cells (Ctr). Error bars: standard deviation from independent experiments in duplicate. Isotype control (ctr) as negative control.

Free NO is an inhibitor of the proliferation of myeloblastic leukemia cells and an inducer of monocytic differentiation [40,41,42]. Therefore, the antiproliferative effect of the BGE was also investigated to verify the activation of cell differentiation pathways. To this aim, the action of BGE was studied on U937 cell expression of CD11c and CD14, markers of granulocyte and monocyte differentiation, respectively. BGE induced maturation-related pathways with consistent monocyte and granulocyte maturation of U937 cells treated with 1.25 and 2.5 mg/mL BGE for 24 h (Figure 6).

Furthermore, the significant increases of free NO and TBARS in the culture medium and cytosol of U937 cells, respectively, observed after treatment with 2.5 mg/mL BGE for 24 h (Figure 2, central and bottom panels), correlated consistently with the expression of CD11c and CD14 markers (Figure 6). The differentiative activity observed in U937 cells after treatment with BGE also correlates with microscopic analysis (Figure 4), and with viability assay (Figure 2, upper pane), which together indicate an evident cell proliferation inhibition. Although further studies are needed, these important effects strongly suggest that the induction of differentiation can contribute to the BGE-mediated anticancer action.

## 4. Conclusions

The great interest in functional foods with strong biomedical activities has stimulated the study of BG for its potential beneficial effects on human health. This work showed the metabolite profile of BGE and its potential benefits on liver cells and on leukemic cell lines. To the best of our knowledge, this is the first study in which the BG crude methanol extract was shown to be able to induce cell differentiation. These aspects are particularly relevant in view of the potential health effects obtained from the consumption of this food. On the other hand, the enrichment in the biologically active compounds by heat aging could have likely increased these benefits and helped to promote the functionalization of garlic. Likely, the biological properties showed by BGE are due to the presence in the extract of pharmacologically important compounds, such as the organosulfur compounds S-allyl-cysteine, alliin and allicin, organic acids, and saponins. However, the exact mechanism of action of all ingredients and their long-term effects are not yet fully understood, so further studies are needed to elucidate these important aspects. Moreover, studies on the human health impact by assessing bioavailability and metabolism have to be carried out. The results of this study will be helpful for the application of BG as a preventive anticancer agent as well as in the prevention and treatment of liver diseases. 

## Figures and Tables

**Table 1:** LC-ESI/HRMS/MS data and putative identification of metabolites in black garlic methanol extract (BGE).

n°	Compound	*R_t_* (min)	Molecular Formula	[M-H]^−^	[(M + H_2_CO_2_)-H]^−^	Delta (ppm)	Characteristic Product Ions (*m/z*)
**1**	S-Allyl-cysteine	1.08	C_6_H_11_NO_2_S	160.0430		2.09	73, 88
**2**	Alliin	1.44	C_6_H_11_NO_3_S	176.0383		3.92	88, 89
**3**	disaccharide	1.49	C_12_H_22_O_11_	341.1090	387.1141	3.44	323, 297, 281, 251, 179, 161, 143, 113
**4**	β-D-fructofuranose 2′,1:2,1′-dianhydride	1.54	C_12_H_20_O_10_	323.0984	369.1038	3.36	233, 161
**5**	Allicin	1.64	C_6_H_10_OS_2_	161.0092		1.47	73, 89
**6**	hexose-phosphate	1.75	C_6_H_13_O_9_P	259.0220		2.53	241, 217, 199, 169, 97, 79
**7**	L-pyroglutamic acid-*O*- hexose ester	1.94	C_11_H_17_O_8_N	290.0876		1.88	272, 230, 200, 170, 128, 113
**8**	Citric acid	2.30	C_6_H_8_O_7_	191.0193		3.51	173, 147, 111, 87
**9**	Methyl citric acid	2.52	C_7_H_10_O_7_	205.0350		3.66	173, 161, 143, 111
**10**	γ-Glutamylphenylalanine	6.21	C_14_H_18_O_5_N_2_	293.1136		1.24	275, 249, 164, 128
**11**	γ-Glutamylphenylalanine-*O*-hexose ester	6.88	C_20_H_28_O_10_N_2_	455.1659		-0.07	437, 393, 365, 335, 293, 290, 275, 200, 170, 164, 128
**12**	Syringin rhamnoside	15.09	C_23_H_34_O_13_	517.1916	517.1916	0.04	499, 307, 163
**13**	Porrigenin B 3-*O*-pentasaccharide	17.55	C_57_H_92_O_30_	1255.5562	1301.5618	-2.24	1093, 931, 769
**14**	9,12,13-Trihydroxy octadecenoic acid	21.30	C_18_H_34_O_5_	329.2329		1.91	311, 293, 229, 211, 199, 197, 171, 141, 127
**15**	gitogenin 3-*O*-tetrasaccharide or β-chlorogenin 3-*O*-tetrasaccharide	23.75	C_51_H_84_O_24_	1079.5248	1125.5314	-1.94	917, 899, 755, 593
**16**	gitogenin 3-*O*-trisaccharide or β-chlorogenin-3-*O*-trisaccharide	24.18	C_45_H_74_O_19_	917.4766	963.4778	2.73	755, 737, 593
**17**	Tigogenin	27.10	C_27_H_44_O_3_	415.3192		-3.49	371, 369, 355, 347, 311, 253
**18**	Hydroxyjasmonic acid sulfate	27.31	C_12_H_18_O_7_S	305.0694		1.38	287, 261, 225, 207, 175, 97
**19**	DGMG (18:3)	28.54	C_33_H_56_O_14_	675.3573	721.3636	-1.97	397, 277
**20**	l-PE (18:2)	30.80	C_23_H_44_O_7_NP	476.2775		0.64	279, 214, 196, 153
**21**	DGMG (18:2)	30.82	C_33_H_58_O_14_	677.3737	723.3794	-0.83	415, 397
**22**	l-PC (18:2)	31.05	C_26_H_50_O_7_NP		564.3296	0.09	504, 279
**23**	l-PE (18:2)	31.62	C_23_H_44_O_7_NP	476.2769		-0.64	279, 214, 196, 153
**24**	l-PC (18:2)	31.92	C_26_H_50_O_7_NP		564.3294	-0.33	504, 279
**25**	l-PE (16:0)	32.55	C_21_H_44_O_7_NP	452.2779		1.53	255, 214, 196
**26**	l-PE (18:2)	32.60	C_23_H_44_O_7_NP	476.2767		-1.04	279, 214, 196, 153
**27**	DGMG (16:0)	32.61	C_31_H_58_O_14_	653.3752	699.3796	1.37	397
**28**	l-PC (16:0)	32.91	C_24_H_50_O_7_NP		540.3299	0.67	480, 255
**29**	l-PC (18:2)	32.96	C_26_H_50_O_7_NP		564.3296	0.09	504, 279
**30**	l-PE (16:0)	33.73	C_21_H_44_O_7_NP	452.2769		-0.54	255, 214, 196
**31**	l-PC (16:0)	34.20	C_24_H_50_O_7_NP		540.3296	0.10	480, 255
**32**	l-PC (18:1)	35.17	C_26_H_52_O_7_NP		566.3451	-0.06	506, 281
**33**	l-PC (18:1)	36.71	C_26_H_52_O_7_NP		566.3450	0.06	506, 281
**34**	l-PI (18:2)	50.45	C_27_H_49_O_12_P	595.2869		-1.34	415, 315, 279, 241
**35**	l-PI (18:2)	52.91	C_27_H_49_O_12_P	595.2877		-0.12	415, 333, 315, 279, 241, 223
**36**	l-PI (16:0)	55.17	C_27_H_49_O_12_P	571.2873		-0.77	409, 391, 315, 255, 241, 223, 171
**37**	l-PI (18:2)	55.73	C_27_H_49_O_12_P	595.2874		-0.64	415, 315, 279, 241, 171
**38**	l-PI (16:0)	58.76	C_25_H_49_O_12_P	571.2880		0.40	409, 391, 315, 255, 241, 223, 171

Legend: DGMG, di-galactosylmonoacylglycerol; l-PE, lyso-phosphatidylethanolamine; l-PC, lyso-phosphatidylcholine; l-PI lysophosphatidylinositol.

**Table 2:** Glucose, cholesterol (CHO), and triglycerides (TG) concentration in the culture medium of HG-HepG2 cells treated for 72 h with 1.0 mg/mL BGE compared to untreated control cells (CTR). The TBARS were evaluated in HG-HepG2 cells after 72 h of 1.0 mg/mL BGE treatment.

	Glucose (mg/dL/Cell n.)	CHO (mg/dL/Cell n.)	TG (mg/dL/Cell n.)	TBARS (M/g/L)
HG-HepG2 (CTR)	638 ± 50	10 ± 1	15 ± 3	0.15 ± 0.03
HG-HepG2 + BGE	1530 ± 200	38 ± 6	74 ± 7	0.098 ± 0.005

## Data Availability

Not necessary.
